# Anti-inflammatory and renoprotective effects of difelikefalin, a kappa opioid receptor agonist, in a rat model of renal ischemia–reperfusion-induced acute kidney injury

**DOI:** 10.1186/s12882-025-04199-9

**Published:** 2025-07-01

**Authors:** Hiroto Takeuchi, Satoshi Tatemichi, Yuji Okuhara

**Affiliations:** 1 Pharmacology Research Laboratory, Kissei Pharmaceutical Co., Ltd., 4365-1 Kashiwabara, Hotaka, Azumino, Nagano 399-8304 Japan; 2 Safety Research Department, Kissei Pharmaceutical Co., Ltd., 2320-1 Maki, Hotaka, Azumino, Nagano 399-8305 Japan

**Keywords:** Renal ischemia–reperfusion injury, Kappa opioid receptor, Difelikefalin, Nalfurafine hydrochloride, Cytokine

## Abstract

**Background:**

This study evaluated the effects of difelikefalin, a kappa opioid receptor (KOR) agonist, on the inflammatory response and renal dysfunction in a rat model of acute kidney injury induced by renal ischemia–reperfusion injury, comparing it with other KOR agonists.

**Methods:**

One week after right nephrectomy, rats received one of three drugs (difelikefalin, nalfurafine hydrochloride, or U-50488H). Thirty minutes later, ischemia was induced in the left kidney by clamping its artery and vein for 45 min. Rats were then transferred to metabolic cages for 24-h urine collection, and blood was taken at the 24-h time point. Renal function (blood urea nitrogen [BUN], serum creatinine [SCr], and creatinine clearance [CCr]) and 23 serum cytokines were assessed, along with histopathological evaluation of renal tubular damage.

**Results:**

Compared with the sham group, the vehicle group showed increased BUN and SCr levels, reduced CCr, and renal tubular damage. Difelikefalin improved BUN, SCr, and CCr levels and mitigated renal tubular damage. Similar effects were observed with nalfurafine hydrochloride and U-50488H. Thirteen serum cytokines were elevated in the vehicle group. Difelikefalin suppressed all elevated cytokines, U-50488H showed moderate efficacy, and nalfurafine hydrochloride exhibited little inhibitory effect.

**Conclusion:**

These findings indicate that difelikefalin has anti-inflammatory and renoprotective effects in a rat acute kidney injury model, with varying anti-inflammatory efficacy among KOR agonists.

**Supplementary Information:**

The online version contains supplementary material available at 10.1186/s12882-025-04199-9.

## Background

Renal ischemia–reperfusion (I/R) injury is a major cause of acute kidney injury (AKI) and occurs in various clinical settings such as surgery, trauma, and hypovolemic shock [1, 2]. I/R injury involves a temporary cessation of blood flow to the kidneys followed by rapid restoration, leading to tissue damage and inflammation [3]. This process promotes the production of inflammatory cytokines, resulting in structural and functional damage to renal tissue [2–4]. AKI is characterized by a rapid decline in kidney function over a short period. Although it was once considered completely reversible, recent studies suggest that AKI may progress to chronic kidney disease (CKD) [5]. CKD involves irreversible damage to kidney structure and fibrosis and is associated with increased cardiovascular disease and mortality rates [6]. Recent research indicates that the interaction between AKI and CKD can significantly alter kidney pathophysiology [7].

Patients with CKD often experience severe pruritus. In addition, severe pruritus associated with hemodialysis is considered a systemic inflammatory disease rather than a local skin disease, and it is associated with sequelae that cause life-threatening complications with an increased risk of death. Indeed, in patients undergoing hemodialysis, various cytokines in plasma can reportedly cause inflammatory conditions and contribute to increased dialysis-related mortality; such cytokines include interleukin (IL)-1, tumor necrosis factor-α (TNF-α), IL-6, and others [8–10].

Difelikefalin (CR845, Korsuva™; Cara Therapeutics, Stamford, CT, USA) is a selective kappa opioid receptor (KOR) agonist designed to limit migration to the central nervous system to regulate pruritus, visceral and inflammatory pain, and inflammatory signals and to reduce the occurrence of insomnia by acting primarily on KORs in the peripheral and immune systems [11–13]. It is currently used in Europe, the United States, and Japan as a treatment for pruritus in patients undergoing hemodialysis.

Interestingly, apart from its antipruritic effects, U-50488H, another KOR agonist, was recently reported to exhibit renoprotective effects in a rat model of renal I/R-induced AKI [14]. This model has been widely used to evaluate the efficacy of drugs for renal damage. In addition to these changes, elevations of inflammatory cytokines in the blood and various organs (liver, kidney, heart, intestine, and brain) are also induced in this model [15]. Therefore, it is suggested that this model is useful not only for evaluating the presence or absence of renoprotective action but also for evaluating anti-(systemic) inflammatory action. No studies have focused on the effects of difelikefalin using this model, and whether difelikefalin has anti-inflammatory and renoprotective effects remains unclear. Therefore, the present study was performed to clarify the possible inflammatory signal-modulating effect and renoprotective effects of difelikefalin using this model and to compare these effects with those of other KOR agonists, including nalfurafine hydrochloride (TRK-820, Remitch^®^; Toray Industries, Inc., Tokyo, Japan), which is approved in Japan for the treatment of dialysis-related pruritus [13], and U-50488H.

## Methods

### Drugs

The test drugs used in this study were as follows: difelikefalin acetate (Maruishi Pharmaceutical Co., Ltd., Osaka, Japan), nalfurafine hydrochloride (HY-12745A; MedChemExpress LLC, Monmouth Junction, NJ, USA), U-50488H (U111; Sigma-Aldrich Chemical Company, St. Louis, MO, USA), and nor-binaltorphimine (nor-BNI) (ab120078; Abcam, Cambridge, UK). All drugs were dissolved and diluted in saline. The dosing solution of difelikefalin acetate was prepared in its active form, difelikefalin, and that of nor-BNI was prepared as its anhydride derivative.

### Animals

This study was performed in accordance with our institutional guidelines, which conform to the Guide for the Care and Use of Laboratory Animals (Eighth Edition) and current Japanese regulations. The study was approved by the Laboratory Animal Care and Use Committee of the Central Research Laboratories of Kissei Pharmaceutical Co., Ltd. (Committee approval numbers: 22A-006, 22B-043). Male Sprague–Dawley rats (The Jackson Laboratory Japan, Inc., Yokohama, Japan), aged 5–7 weeks and weighing 110–225 g upon arrival, were housed in groups of five per cage and maintained in an environment with a constant temperature of 21.0–24.9 °C and humidity ranging from 41% to 67%. They were subjected to a 12-h light (08:00–20:00)/12-h dark cycle. All animals had free access to water and a standard diet of CE-2 pellets (CLEA Japan, Inc., Tokyo, Japan).

### Experimental design

**Fig. 1:**
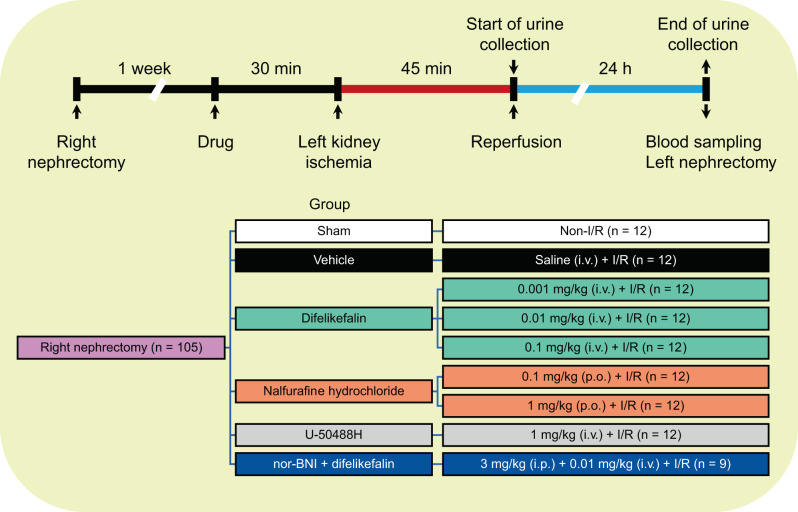
Scheme of the experiment. I/R: ischemia–reperfusion, nor-BNI: nor-binaltorphimine, i.v.: intravenously, p.o.: per os, i.p.: intraperitoneally

The experimental design is illustrated in Fig. 1. The rats were allocated to 9 groups (8 groups consisting of 12 rats each, and 1 group consisting of 9 rats). The groups were assigned as follows: a sham group (non-I/R), a vehicle group (saline + I/R), three difelikefalin groups (difelikefalin [0.001, 0.01, and 0.1 mg/kg] + I/R), two nalfurafine hydrochloride groups (nalfurafine hydrochloride [0.1 and 1 mg/kg] + I/R), a U-50488H group (U-50488H [1 mg/kg] + I/R), and a nor-BNI + difelikefalin group (nor-BNI [3 mg/kg] + difelikefalin [0.01 mg/kg] + I/R).

### I/R rat models and experimental protocol

The experimental procedure was conducted with reference to past reports [14–16]. Strict aseptic techniques were adhered to during all surgical procedures. Under anesthesia induced with 2% isoflurane inhalation (Mylan EPD G.K., Osaka, Japan) and maintained on a heating pad set to 37 °C, an incision was made on the right side of the abdomen of 8-week-old rats. The right kidney was removed following ligation of the renal artery, renal vein, and ureter using sterile silk suture. Postoperatively, the surgical site was sprayed with a 0.5% solution of kanamycin sulfate (FUJIFILM Wako Pure Chemical Corporation, Osaka, Japan). One week after right nephrectomy, left renal ischemia was induced as follows. The rats were anesthetized with 2% isoflurane on a heating pad, and the left kidney was exposed through a small flank incision. The left renal artery and vein were occluded with a non-traumatic clip (Schwartz Micro Serrefine Light Bend; Fine Science Tools Inc., Foster City, CA, USA) for 45 min. When the color of the kidney changed from bright red to purple-black, the clipping procedure was determined to be successful. At the end of the ischemic period, the clip was gently removed for reperfusion, which was confirmed by the restoration of blood flow. When the color of the kidney changed from purple-black to bright red, the reperfusion was determined to be successful.

In the difelikefalin groups (0.001, 0.01, and 0.1 mg/kg) and U-50488H group (1 mg/kg), the drugs were intravenously administered into the tail vein 30 min before ischemia. In the nalfurafine hydrochloride groups (0.1 and 1 mg/kg), the drug was orally administered 30 min before ischemia. In the KOR agonist and KOR antagonist groups, nor-BNI (3 mg/kg) was intraperitoneally administered 30 min before administration of difelikefalin (0.01 mg/kg intravenously). In the vehicle group, saline was injected into the tail vein 30 min before ischemia. In the sham group, the same surgical procedures described above were performed without occlusion of the renal artery and vein. The incision was then closed with sterile silk suture, as for right nephrectomy. After waking from anesthesia, the rats were individually placed in metabolic cages and supplied with food and water for 24 h after I/R surgery. Twenty-four-hour urine samples were collected, and blood samples were taken from the cervical vein at the end of the urine collection period.

After the urine and blood samples had been collected, the animals were fully anesthetized with 3% isoflurane. Following confirmation that the anesthesia was sufficient, the animals were euthanized by performing a bilateral thoracotomy to expose the heart, inserting a cannula into the heart, and perfusing with saline (approximately 100 mL). The left kidneys were removed and fixed in 10% neutral buffered formalin solution (Mildform 10 N; FUJIFILM Wako Pure Chemical Corporation, Osaka, Japan).

### Detection of urea nitrogen and creatinine

Inhalation of 2% isoflurane was used to anesthetize the rats 24 h after the I/R surgery, and cervical vein puncture was performed to collect 1.2–1.4 mL of blood. After 0.5–1.0 h of preservation at room temperature to ensure coagulation, the blood samples were centrifuged for 5 min at 1000 × *g* (25 °C), and the upper layer of serum was collected. The collected urine samples were centrifuged for 5 min at 1000 × *g* (25 °C), and the urine volume was measured. An automated biochemical analyzer (Fuji DRI-CHEM 7000VZ; FUJIFILM Medical Co., Ltd., Tokyo, Japan) was used to detect the levels of blood urea nitrogen (BUN) and serum creatinine (SCr). The remaining serum samples were stored at −80 °C until cytokine measurement. Urine creatinine (UCr) was detected using LabAssay™ Creatinine (FUJIFILM Wako Shibayagi Corporation, Gunma, Japan). After the reaction, the absorbance at the 520-nm wavelength was measured with a SpectraMax^®^ 190 Absorbance Plate Reader (Molecular Devices Japan K.K., Tokyo, Japan), and the UCr concentration was calculated. The creatinine clearance (CCr) was then calculated (UCr/SCr × urine flow/body weight).

### Histopathological examination

Formalin-fixed kidneys from all animals were sectioned longitudinally in the middle, including the renal papilla, to ensure consistency in the observation plane across all animals. The tissues were embedded in paraffin, cut into 3-µm-thick sections, and stained with periodic acid–Schiff. For each kidney tissue section, approximately 50 microscopic fields (actual number of fields: 36–50 per animal) from the cortex to the outer medulla were observed at × 200 magnification using a microscope (BX-53; Olympus, Tokyo, Japan), and the renal tubule with the most severe damage in each field was scored. The scoring method was in accordance with the Paller scoring method [17]. Each renal tubule was scored as follows: the flatness of the epithelial cells ranged from 0 to 2 points, cytoplasm vacuolization ranged from 0 to 1 point, brush margin loss ranged from 0 to 1 point, cell necrosis ranged from 0 to 2 points, bleeding ranged from 0 to 2 points, interstitial edema ranged from 0 to 1 point, and luminal obstruction ranged from 0 to 2 points. A total histopathological score of the observed microscopic field was calculated by combining the scores for each of the above parameters, resulting in a maximum score of 9 points. Differences between groups were statistically evaluated by calculating the mean score of the observed fields for each animal and determining the mean score for each group (*n* = 12/group).

### Measurement of serum cytokine levels

The following 23 cytokines (interleukins, chemokines, and growth factors) in the serum of rats were detected using a Bio-Plex Pro™ Rat Cytokine 23-Plex Assay (Bio-Rad Laboratories, Hercules, CA, USA): IL-1α, IL-1β, IL-2, IL-4, IL-5, IL-6, IL-7, IL-10, IL-12 (p70), IL-13, IL-17, IL-18, granulocyte colony-stimulating factor (G-CSF, also known as CSF3), macrophage colony-stimulating factor (M-CSF), granulocyte-macrophage colony-stimulating factor (GM-CSF), interferon-γ (IFN-γ), TNF-α, growth-related oncogene/keratinocyte-derived chemokine (GRO/KC, also known as CXCL1), monocyte chemoattractant protein-1 (MCP-1, also known as CCL2), macrophage inflammatory protein-1α (MIP-1α, also known as CCL3), MIP-3α (also known as CCL20), regulated upon activation in normal T cells expressed and secreted (RANTES, also known as CCL5), and vascular endothelial growth factor (VEGF). All samples were analyzed in accordance with the manufacturer’s protocol. The analyte concentrations are expressed in pg/mL.

### Statistical analysis

Data are presented as mean ± standard error of the mean. Statistical analyses were performed using SAS Version 9.3 (SAS Institute, Cary, NC, USA) and its connected program EXSUS Version 10.0.6 (EPS Corporation, Tokyo, Japan). Data were analyzed among the vehicle group and the drug-treated groups as follows. When equality of variance was indicated by Bartlett’s test, the statistical analysis was performed using Dunnett’s test. When equality of variance was not indicated by Bartlett’s test, the statistical analysis was performed using Steel’s test. Comparisons between the sham group and the vehicle group, as well as for renal function evaluation between the difelikefalin (0.01 mg/kg) and nor-BNI + difelikefalin (0.01 mg/kg) groups, were performed as follows. When equality of variance was indicated by the F-test, the statistical analysis was performed using Student’s t-test. When equality of variance was not indicated, the statistical analysis was performed using the Aspin–Welch t-test. A value of *P* < 0.05 was considered statistically significant.

## Results

### Effects on renal function in rats with I/R injury

**Fig. 2:**
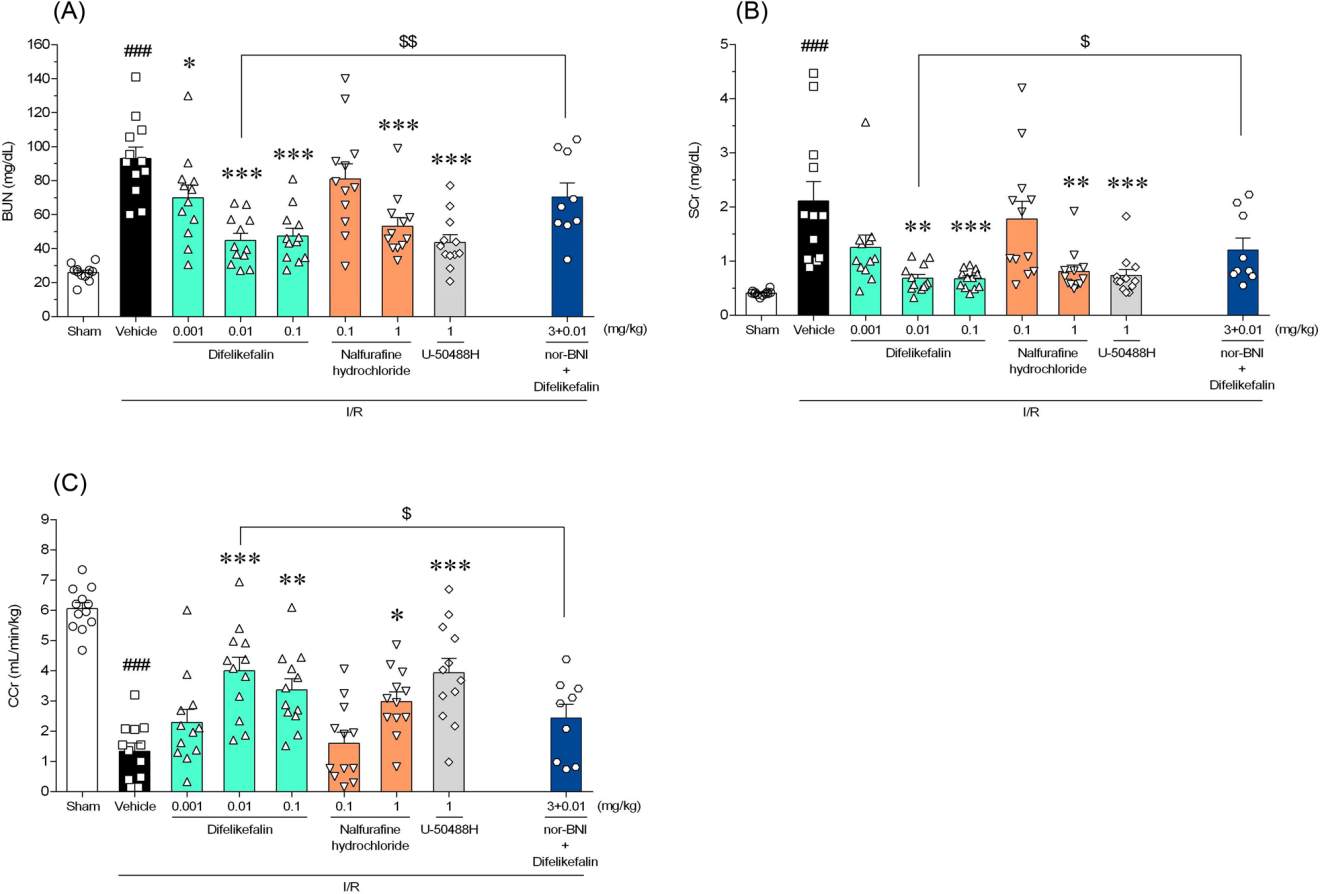
Effects of difelikefalin, nalfurafine hydrochloride, and U-50488H on biochemical parameters. **A** BUN, **B** SCr, and **C** CCr were measured or calculated 24 h after surgical establishment of renal I/R injury. In addition to the effects of KOR agonists, the inhibitory effect of the KOR antagonist nor-BNI on the effect of difelikefalin was evaluated. nor-BNI (3 mg/kg, i.p.) was administered 30 min before difelikefalin (0.01 mg/kg, i.v.). Each column and bar represents the mean ± standard error of the mean in 12 rats (nor-BNI + difelikefalin group: 9 rats). ^###^*P* < 0.001 compared with sham group (Student’s t-test or Aspin–Welch t-test). *P < 0.05, **P < 0.01, ***P < 0.001 compared with vehicle group (Dunnett’s test or Steel’s test). ^$^*P* < 0.05, ^$$^*P* < 0.01 compared with difelikefalin group (0.01 mg/kg) (Student’s t-test or Aspin–Welch t-test). BUN: blood urea nitrogen, SCr: serum creatinine, CCr: creatinine clearance, nor-BNI: nor-binaltorphimine

The BUN and SCr levels were significantly higher in the serum of the vehicle group than the sham group. Difelikefalin (0.01 and 0.1 mg/kg intravenously) significantly suppressed the elevation of the BUN and SCr levels in the rats with I/R injury. CCr was reduced in the vehicle group, and this reduction was reversed by 0.01 and 0.1 mg/kg difelikefalin. Similar to difelikefalin, nalfurafine hydrochloride (1 mg/kg orally) and U-50488H (1 mg/kg intravenously) also significantly suppressed the increase in BUN and SCr and the decrease in CCr. The KOR antagonist nor-BNI (3 mg/kg intraperitoneally) showed antagonistic effects on the suppression of renal dysfunction by difelikefalin (0.01 mg/kg intravenously) (Fig. 2).

### Effects on histopathological changes of renal tissue in rats with I/R injury

**Fig. 3:**
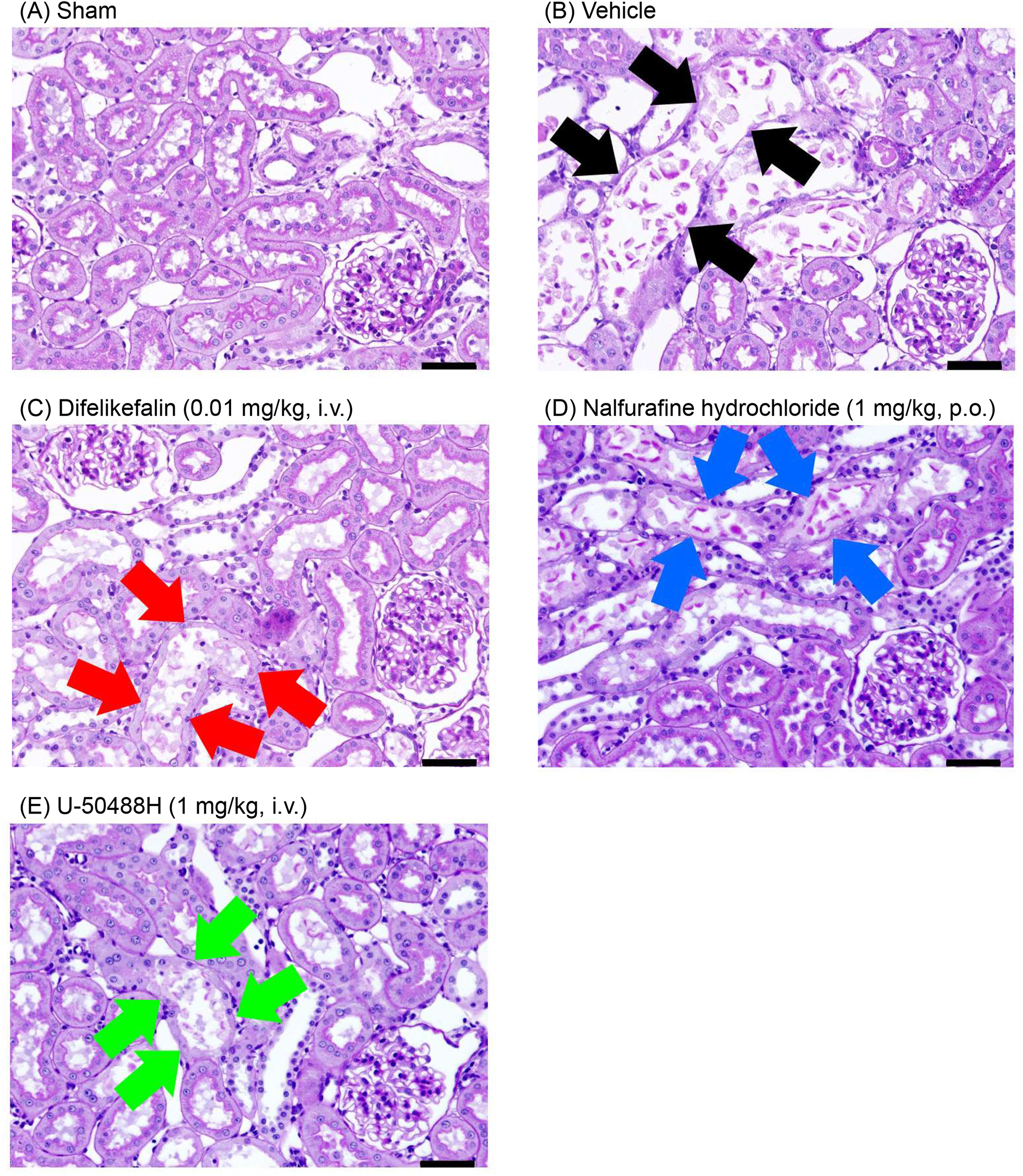
Representative images of renal tissues with periodic acid–Schiff staining. Left kidneys extracted 1 day after the I/R procedure in rats were microscopically observed. **A** Sham group: normal tubular epithelial cells. **B** Vehicle group: severe necrosis and loss of the brush border of tubular epithelial cells with moderate luminal obstruction (black arrows). **C** Difelikefalin (0.01 mg/kg, i.v.) group: slight necrosis and loss of the brush border of the tubular epithelial cells with moderate luminal obstruction (red arrows). **D** Nalfurafine hydrochloride (1 mg/kg, p.o.) group: severe necrosis and loss of the brush border of tubular epithelial cells with slight luminal obstruction (blue arrows). **E** U-50488H (1 mg/kg, i.v.) group: slight necrosis and loss of the brush border of tubular epithelial cells with moderate luminal obstruction (green arrows). Scale bar = 50 μm

**Fig. 4:**
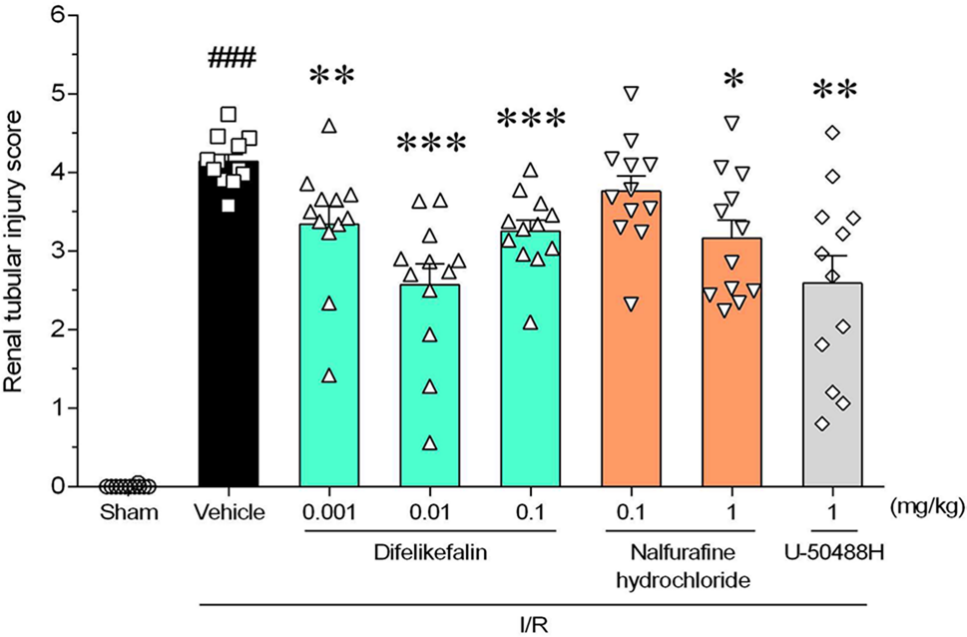
Effects of difelikefalin, nalfurafine hydrochloride, and U-50488H on renal tubular injury. Histopathological scoring of renal tubular injury of kidneys extracted 1 day after I/R procedure. See Methods for scoring criteria. Each column and bar represents the mean ± standard error of the mean in 12 rats. ^###^*P* < 0.001 compared with sham group (Aspin–Welch t-test). *P < 0.05, **P < 0.01, ***P < 0.001 compared with vehicle group (Steel’s test)

Pathologic assessments were performed in all groups except the antagonist treatment group. The histopathologic examination in the vehicle group revealed severe destruction of renal tubular cells, including severe necrosis of the tubular epithelial cells and luminal obstruction. However, the difelikefalin group showed a relatively well-protected structure compared with the vehicle group (Fig. 3). The tubular injury score was significantly lower in the difelikefalin groups (0.001, 0.01, and 0.1 mg/kg intravenously), nalfurafine hydrochloride group (1 mg/kg orally), and U-50488H group (1 mg/kg intravenously) than in the vehicle group (Fig. 4).

### Effects on cytokines in rats with I/R injury

**Fig. 5:**
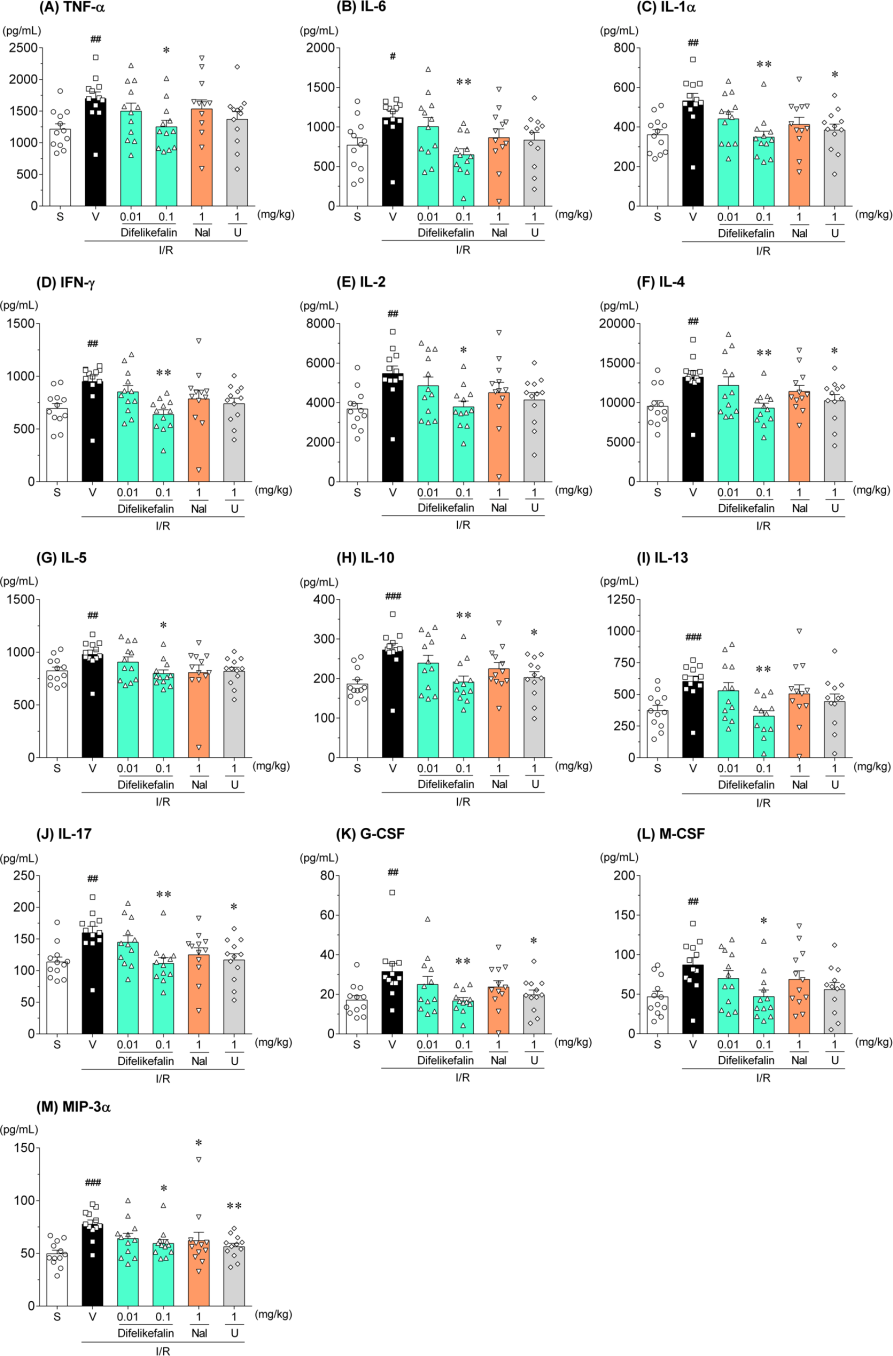
Effects of difelikefalin, nalfurafine hydrochloride, and U-50488H on serum cytokines. Cytokines in rat serum collected 24 h after surgical establishment of renal I/R injury were measured. **A** TNF-α, **B** IL-6, **C** IL-1α, **D** IFN-γ, **E** IL-2, **F** IL-4, **G** IL-5, **H** IL-10, **I** IL-13, **J** IL-17, **K** G-CSF, **L** M-CSF, and **M** MIP-3α were higher in the serum of the vehicle group than sham group. In each bar chart, the concentrations of serum cytokines in the drug treatment group were compared with those in the vehicle group. Each column and bar represents the mean ± standard error of the mean in 12 rats. S: sham, V: vehicle, Nal: nalfurafine hydrochloride, U: U-50488H. ^#^*P* < 0.05, ^##^*P* < 0.01, ^###^*P* < 0.001 compared with sham group (Student’s t-test). *P < 0.05, **P < 0.01 compared with vehicle group (Dunnett’s test or Steel’s test). TNF: tumor necrosis factor, IL: interleukin, IFN: interferon, G-CSF: granulocyte colony-stimulating factor, M-CSF: macrophage colony-stimulating factor, MIP: macrophage inflammatory protein

Serum cytokines in the sham group and vehicle group, as well as those in the drug treatment group that showed efficacy against reduced renal function, were measured. Of the 23 inflammatory cytokines and other cytokines, 13 (TNF-α, IL-6, IL-1α, IFN-γ, IL-2, IL-4, IL-5, IL-10, IL-13, IL-17, G-CSF, M-CSF, and MIP-3α) were significantly higher in the serum of the vehicle group than the sham group (Fig. 5). However, the remaining 10 cytokines, including inflammatory cytokines such as IL-1β, MCP-1, and IL-12, did not differ between the two groups (Additional file 1). Difelikefalin (0.1 mg/kg intravenously) significantly suppressed all elevated cytokines to the same level as in the sham group. U-50488H exhibited moderate effects. By contrast, nalfurafine hydrochloride had no inhibitory effect except for MIP-3α (Fig. 5).

## Discussion

In the present study of renal I/R-induced AKI in rats, the KOR agonist difelikefalin suppressed the elevation of blood inflammatory cytokines and other cytokines 24 h after I/R and showed inhibitory effects on renal function decline and histological renal damage.

The renal I/R-induced AKI model used in this study was not a CKD model. However, AKI is reportedly a risk factor for CKD [5, 18], and the renal I/R-induced AKI model used in this study has been shown to cause distant organ damage in the heart, lungs, brain, gut, and liver [1, 5, 15]. Although the precise mechanisms by which AKI causes pathological changes in distant organs have not been elucidated, the production of inflammatory cytokines and chemokines in the blood and each organ caused by AKI, the migration of leukocytes, the production of oxidative stress, and the metabolic dysregulation of electrolytes and water have been shown to be important or common factors in the damage of each organ [15, 19].

In this study, 13 of the 23 cytokines were elevated in the vehicle group 24 h after renal I/R, including inflammatory cytokines such as TNF-α, IL-6, IL-1α, and IL-2. Although this model showed elevated levels of inflammatory cytokines such as TNF-α, IL-6, and IL-1β, there are few detailed reports on other cytokine changes and the effects of KOR agonists on changes in these inflammatory mediators [20–22]. Therefore, the results obtained in this study may be useful as new findings for this model. We were unable to confirm the elevations of IL-1β and MCP-1, which have been reported to increase in this model, but the reason for this discrepancy was considered to be the differences in the experimental conditions (e.g., ischemia method, ischemia time, blood collection point) [20, 21, 23, 24].

In this study, difelikefalin suppressed all the elevated blood cytokines, and U-50488H showed a moderate effect. KOR is expressed in the central and peripheral nervous systems [25] as well as in the kidney [26, 27] and immune cells [28], and U-50488H can reportedly reduce the levels of inflammatory cytokines such as IL-1 and TNF-α [22]. Although the mechanism of action of KOR agonists on elevated cytokines and renal dysfunction is not clear, the involvement of the PI3K/Akt signaling pathway has been suggested [14, 29]. Therefore, we speculate that difelikefalin acts on KOR of the kidney and immune cells, and such a mechanism of action likely enables it to suppress cytokine elevations and regulate inflammatory signaling. This inhibitory effect of difelikefalin may contribute to the elevation of inflammatory cytokines, part of the pathogenesis of CKD in patients undergoing hemodialysis.

Interestingly, nalfurafine hydrochloride did not have a clear inhibitory effect on cytokine elevations, differing from difelikefalin and U-50488H. The reason for this discrepancy is unknown, but possible causes include differences in pharmacokinetics (metabolism, migration) due to differences in the routes of administration (intravenous for difelikefalin and U-50488H vs. oral for nalfurafine hydrochloride) as well as differences in ligand recognition mechanisms [30].

In this study, we found that difelikefalin suppressed renal damage in addition to exhibiting anti-inflammatory effects. Nalfurafine hydrochloride used as a comparative agent showed a similar inhibitory effect on renal damage, and U-50488H showed similar results to those described in previous reports [14]. In addition, we confirmed that a KOR antagonist attenuated the protective effect of difelikefalin on renal function. These results suggest that KOR agonists have a renoprotective effect.

Regarding whether the anti-inflammatory and renoprotective effects of difelikefalin are KOR-mediated, investigations using a KOR antagonist have only focused on renal function and did not study pathological assessment and cytokines. However, a correlation has been confirmed between renal function parameters and tubular injury scores (data not shown), and the inhibitory effects of U-50488H on renal damage and inflammatory cytokines have been reported to be reversed by a KOR antagonist [14, 22]. Therefore, we consider these effects to be mediated by the KOR. In addition, the doses at which difelikefalin showed efficacy in this model (0.01 and 0.1 mg/kg intravenously) were not different from those in a pruritus model [11, 12], suggesting that difelikefalin may exert anti-inflammatory and renoprotective effects at doses similar to those that induce antipruritic effects.

Difelikefalin (0.01 mg/kg intravenously) and nalfurafine hydrochloride (1 mg/kg orally) showed inhibitory effects on renal damage without obvious anti-inflammatory effects, but the degree was not different from that of difelikefalin (0.1 mg/kg intravenously) and U-50488H (1 mg/kg intravenously), which showed both anti-inflammatory effects and inhibitory effects on renal damage. These results suggest that KOR agonists may exhibit renoprotective effects in this model regardless of whether the drugs have anti-inflammatory effects.

Although the target population of these drugs is patients with renal function decline undergoing dialysis, restoring renal function is difficult. Nevertheless, maintaining residual renal function in patients undergoing dialysis is important because it reportedly influences the effectiveness of dialysis therapy [31–33]. Therefore, KOR agonists, which have renoprotective action, are expected to contribute to improving the life prognosis of patients undergoing dialysis.

Regarding the anti-pruritic effect of the difference in anti-inflammatory activity between difelikefalin and nalfurafine hydrochloride, investigation into the relationship between pruritus and inflammation has revealed the following. First, patients undergoing hemodialysis who develop pruritus with no obvious visible signs of inflammation have significantly higher serum levels of C-reactive protein, IL-6, IFN-γ [34], IL-2 [35], and IL-31 [36] than patients without pruritus. Second, in patients with pruritus undergoing hemodialysis, persistent and systemic itching induces scratching, which causes the release of inflammatory cytokines and worsens the skin condition. The deterioration of the skin condition further enhances the itching. This condition is considered the so-called “vicious circle of itching and scratching” [34, 37]. Therefore, the anti-inflammatory effect of difelikefalin seems to contribute to pruritus to a certain degree.

To more clearly evaluate the effect of difelikefalin on pruritus and the difference in its effect from nalfurafine hydrochloride, it is necessary to study a model that allows for the simultaneous and long-term assessment of renal impairment, inflammation, and pruritus. To the best of our knowledge, however, such a model has not been reported to date. In addition, evaluation is limited because this study used a single-dose administration, and the renal I/R-induced AKI model reportedly recovers about 1 week after reperfusion [16, 38].

In the future, we would like to establish a CKD-associated pruritus model that can be extrapolated to the clinical setting, evaluate the antipruritic effect of difelikefalin and its effects on the kidneys and other organs, and investigate the contribution of its anti-inflammatory effect. By examining the effects of inflammation on multiple organ damage, we hope that difelikefalin, which has both anti-inflammatory and renoprotective effects in addition to antipruritic effects, may be effective against systemic inflammation. We also hope to determine whether its anti-inflammatory effects play crucial roles in the effect on pruritus and the maintenance of residual renal function.

This study has some limitations. First, the histopathological scoring was performed using single-slide analysis, which may not have fully captured the variability in tissue pathology. Second, the study lacks tissue-level cytokine data, which could provide more insight into the localized inflammatory responses and mechanisms.

## Conclusion

The antipruritic drug difelikefalin, a selective KOR agonist, showed an inhibitory effect on the inflammatory response and renal dysfunction in a rat model of I/R-induced AKI, with varying efficacy in terms of anti-inflammatory action among KOR agonists. The study findings suggest that difelikefalin has an anti-inflammatory effect in addition to a renoprotective effect.

## Electronic supplementary material

Below is the link to the electronic supplementary material.

Supplementary Material 1

## Data Availability

No datasets were generated or analysed during the current study.

## Abbreviations

KOR: Kappa opioid receptor
BUN: Blood urea nitrogen
SCr: Serum creatinine
CCr: Creatinine clearance
CKD: Chronic kidney disease
IL: Interleukin
TNF: Tumor necrosis factor
I/R: Ischemia–reperfusion
AKI: Acute kidney injury
nor-BNI: Nor-binaltorphimine
UCr: Urine creatinine
G-CSF: Granulocyte colony-stimulating factor
M-CSF: Macrophage colony-stimulating factor
GM-CSF: Granulocyte-macrophage colony-stimulating factor
IFN: Interferon
GRO/KC: Growth-related oncogene/keratinocyte-derived chemokine
MCP: Monocyte chemoattractant protein
MIP: Macrophage inflammatory protein
RANTES: Regulated upon activation in normal T cells expressed and secreted
VEGF: Vascular endothelial growth factor

## References

[1] Hashemi SS, Janfeshan S, Karimi Z. Acute lung injury induced by acute uremia and renal ischemic-reperfusion injury: the role of toll-like receptors 2 and 4, and oxidative stress. Iran J Basic Med Sci. 2022;25(5):643–51. 35911649 10.22038/IJBMS.2022.64025.14099 PMC9282739

[2] Karimi Z, SoukhakLari R, Rahimi-Jaberi K, Esmaili Z, Moosavi M. Nanomicellar curcuminoids attenuates renal ischemia/reperfusion injury in rat through prevention of apoptosis and downregulation of MAPKs pathways. Mol Biol Rep. 2021;48(2):1735–43. 33606150 10.1007/s11033-021-06214-2

[3] Karimi Z, Asadi K, Ghahramani P, Gholami A. Trinitroglycerine-loaded chitosan nanoparticles attenuate renal ischemia-reperfusion injury by modulating oxidative stress. Sci Rep. 2024;14(1):32112. 39738455 10.1038/s41598-024-83886-3 PMC11685805

[4] Gholampour F, Roozbeh J, Janfeshan S, Karimi Z. Remote ischemic per-conditioning protects against renal ischemia-reperfusion injury via suppressing gene expression of TLR4 and TNF-alpha in rat model. Can J Physiol Pharmacol. 2019;97(2):112–19. 30501397 10.1139/cjpp-2018-0543

[5] Coca SG, Singanamala S, Parikh CR. Chronic kidney disease after acute kidney injury: a systematic review and meta-analysis. Kidney Int. 2012;81(5):442–48. 22113526 10.1038/ki.2011.379 PMC3788581

[6] Venkatachalam MA, Griffin KA, Lan R, Geng H, Saikumar P, Bidani AK. Acute kidney injury: a springboard for progression in chronic kidney disease. Am J Physiol Renal Physiol. 2010;298(5):F1078–94. 20200097 10.1152/ajprenal.00017.2010 PMC2867413

[7] Chawla LS, Kimmel PL. Acute kidney injury and chronic kidney disease: an integrated clinical syndrome. Kidney Int. 2012;82(5):516–24. 22673882 10.1038/ki.2012.208

[8] Sharif MR, Chitsazian Z, Moosavian M, Raygan F, Nikoueinejad H, Sharif AR, et al. Immune disorders in hemodialysis patients. Iran J Kidney Dis. 2015;9:84–96. 25851286

[9] Elhag S, Rivas N, Tejovath S, Mustaffa N, Deonarine N, Abdullah Hashmi M, et al. Chronic kidney disease-associated pruritus: a glance at novel and lesser-known treatments. Cureus 2022;14:e21127. 35036239 10.7759/cureus.21127 PMC8752116

[10] Cohen SD, Phillips TM, Khetpal P, Kimmel PL. Cytokine patterns and survival in haemodialysis patients. Nephrol Dial Transplant. 2010;25(4):1239–43. 20007982 10.1093/ndt/gfp625

[11] Lipman ZM, Yosipovitch G. An evaluation of difelikefalin as a treatment option for moderate-to-severe pruritus in end stage renal disease. Expert Opin Pharmacother. 2021;22(5):549–55. 33190563 10.1080/14656566.2020.1849142

[12] Momotani K, Nojiri R, Uchiyama T, Taniguchi T. Pharmacological, pharmacokinetic and clinical profiles of Difelikefalin (KORSUVA() IV Injection Syringe for Dialysis), a peripheral kappa opioid receptor agonist. Nihon Yakurigaku Zasshi. 2025;160(2):127–40. 40024699 10.1254/fpj.24050

[13] Inan S, Dun NJ, Cowan A. Antipruritic effect of nalbuphine, a kappa opioid receptor agonist, in mice: a pan antipruritic. Molecules. 2021;26(18):5517. 34576988 10.3390/molecules26185517 PMC8466557

[14] Liu LJ, Yu JJ, Xu XL. Kappa-opioid receptor agonist U50448H protects against renal ischemia-reperfusion injury in rats via activating the PI3K/Akt signaling pathway. Acta Pharmacol Sin. 2018;39(1):97–106. 28770825 10.1038/aps.2017.51 PMC5758666

[15] Shang Y, Madduma Hewage S, Wijerathne CUB, Siow YL, Isaak CK, Karmin O. Kidney Ischemia-Reperfusion elicits acute liver injury and inflammatory response. Front Med Lausanne. 2020;7:201. 32582723 10.3389/fmed.2020.00201 PMC7280447

[16] Matsumura Y, Nishiura M, Deguchi S, Hashimoto N, Ogawa T, Seo R. Protective effect of FK409, a spontaneous nitric oxide releaser, on ischemic acute renal failure in rats. J Pharmacol Exp Ther. 1998;287(3):1084–91. 9864296

[17] Paller MS, Hoidal JR, Ferris TF. Oxygen free radicals in ischemic acute renal failure in the rat. J Clin Invest. 1984;74:1156–64. 6434591 10.1172/JCI111524 PMC425281

[18] Naoki K, Tamaki S. Acute kidney injury: progress in diagnosis and treatments. Topics: IV. Pathophysiology and treatments; 4. Association of CKD and acute kidney injury. Nihon Naika Gakkai Zasshi. 2014;103(5):1094–100. 25026779 10.2169/naika.103.1094

[19] Yap SC, Lee HT. Acute kidney injury and extrarenal organ dysfunction: new concepts and experimental evidence. Anesthesiology. 2012;116(5):1139–48. 22415388 10.1097/ALN.0b013e31824f951b

[20] Zhu J, Qiu JG, Xu WT, Ma HX, Jiang K. Alamandine protects against renal ischaemia-reperfusion injury in rats via inhibiting oxidative stress. J Pharm Pharmacol. 2021;73(11):1491–502. 34244746 10.1093/jpp/rgab091

[21] Fawzy MA, Maher SA, Bakkar SM, El-Rehany MA, Fathy M. Pantoprazole attenuates MAPK (ERK1/2, JNK, p38)-NF-κB and apoptosis signaling pathways after renal ischemia/reperfusion injury in rats. Int J Mol Sci. 2021;22(19):10669. 34639009 10.3390/ijms221910669 PMC8508698

[22] Belkowski SM, Alicea C, Eisenstein TK, Adler MW, Rogers TJ. Inhibition of interleukin-1 and tumor necrosis factor-alpha synthesis following treatment of macrophages with the kappa opioid agonist U50,488H. J Pharmacol Exp Ther. 1995;273(3):1491–96. 7791124

[23] Gao J, Zhang D, Yang X, Zhang Y, Li P, Su X. Lysophosphatidic acid and lovastatin might protect kidney in renal I/R injury by downregulating MCP-1 in rat. Ren Fail. 2011;33(8):805–10. 21815729 10.3109/0886022X.2011.601829

[24] Tweij TR, Al-Issa MA, Hamed M, Khaleq MAA, Jasim A, Hadi NR. Pretreatment with Erythropoietin alleviates the renal damage induced by ischemia reperfusion via repression of inflammatory response. Wiad Lek. 2022;75(12):2939–47. 36723307 10.36740/WLek202212108

[25] Snyder LM, Chiang MC, Loeza-Alcocer E, Omori Y, Hachisuka J, Sheahan TD, et al. Kappa opioid receptor distribution and function in primary afferents. Neuron 2018;99:1274–88. 30236284 10.1016/j.neuron.2018.08.044 PMC6300132

[26] Wittert G, Hope P, Pyle D. Tissue distribution of opioid receptor gene expression in the rat. Biochem Biophys Res Commun. 1996;218(3):877–81. 8579608 10.1006/bbrc.1996.0156

[27] Peng J, Sarkar S, Chang SL. Opioid receptor expression in human brain and peripheral tissues using absolute quantitative real-time RT-PCR. Drug Alcohol Depend. 2012;124(3):223–28. 22356890 10.1016/j.drugalcdep.2012.01.013 PMC3366045

[28] Machelska H, Mö C. Immune cell-mediated opioid analgesia. Immunol Lett. 2020;227:48–59. 32814155 10.1016/j.imlet.2020.08.005

[29] Zeng S, Zhong Y, Xiao J, Ji J, Xi J, Wei X, et al. Kappa opioid receptor on pulmonary macrophages and immune function. Transl Perioper Pain Med 2020;7:225–33. 33204767 10.31480/2330-4871/117 PMC7668421

[30] El Daibani A, Paggi JM, Kim K, Laloudakis YD, Popov P, Bernhard SM, et al. Molecular mechanism of biased signaling at the kappa opioid receptor. Nat Commun 2023;14:1338. 36906681 10.1038/s41467-023-37041-7 PMC10008561

[31] Ng TG, Johnson DW, Hawley CM. Is it time to revisit residual renal function in haemodialysis? Nephrology (Carlton). 2007;12(3):209–17. 17498114 10.1111/j.1440-1797.2007.00795.x

[32] Perl J, Bargman JM. The importance of residual kidney function for patients on dialysis: a critical review. Am J Kidney Dis. 2009;53(6):1068–81. 19394737 10.1053/j.ajkd.2009.02.012

[33] Vilar E, Farrington K. Emerging importance of residual renal function in end-stage renal failure. Semin Dial. 2011;24(5):487–94. 21999737 10.1111/j.1525-139X.2011.00968.x

[34] Kimmel M, Alscher DM, Dunst R, Braun N, Machleidt C, Kiefer T, et al. The role of micro-inflammation in the pathogenesis of uraemic pruritus in haemodialysis patients. Nephrol Dial Transplant. 2006;21:749–55. 16249205 10.1093/ndt/gfi204

[35] Fallahzadeh MK, Roozbeh J, Geramizadeh B, Namazi MR. Interleukin-2 serum levels are elevated in patients with uremic pruritus: a novel finding with practical implications. Nephrol Dial Transplant. 2011;26(10):3338–44. 21372257 10.1093/ndt/gfr053

[36] Ko MJ, Peng YS, Chen HY, Hsu SP, Pai MF, Yang JY, et al. Interleukin-31 is associated with uremic pruritus in patients receiving hemodialysis. J Am Acad Dermatol 2014;71:1151–59. 25270263 10.1016/j.jaad.2014.08.004

[37] Rinaldi G. The itch-scratch cycle: a review of the mechanisms. Dermatol Pract Concept. 2019;9(2):90–97. 31106010 10.5826/dpc.0902a03 PMC6502296

[38] Godoy JR, Watson G, Raspante C, Illanes O. An effective mouse model of unilateral renal ischemia-reperfusion injury. J Vis Exp. 2021;173:e62749. 10.3791/62749 34338680

