# Supplementary material for: Anti-inflammatory and renoprotective effects of difelikefalin, a kappa opioid receptor agonist, in a rat model of renal ischemia–reperfusion-induced acute kidney injury
Source: BMC Nephrol. 2025 Jul 1;26:307. doi: 10.1186/s12882-025-04199-9 (PMC12211451; doi:10.1186/s12882-025-04199-9)
Supplement: Supplementary file 1 — Supplementary Material 1 [file 12882_2025_4199_MOESM1_ESM.docx]

**Additional file 1. Serum cytokines levels (10 types) 24 hours after I/R surgery in rats**

| Cytokine (pg/mL) | Sham | Vehicle | Difelikefalin | | Nalfurafine hydrochloride | U-50488H |
| --- | --- | --- | --- | --- | --- | --- |
|  |  |  | 0.01 mg/kg (i.v.) | 0.1 mg/kg (i.v.) | 1 mg/kg (p.o.) | 1 mg/kg (i.v.) |
| IL-1β | 205 ± 65 | 170 ± 44 | 143 ± 25 | 119 ± 18 | 115 ± 14 | 104 ± 7 |
| IL-7 | 318 ± 109 | 257 ± 59 | 220 ± 37 | 182 ± 28 | 182 ± 19 | 152 ± 11* |
| IL-12 | 706 ± 73 | 1293 ± 334 | 1072 ± 219 | 634 ± 66* | 713 ± 79* | 680 ± 58** |
| IL-18 | 6119 ± 864 | 7384 ± 1070 | 6381 ± 1401 | 4958 ± 769* | 4884 ± 582 | 4677 ± 800 |
| GM-CSF | 308 ± 102 | 257 ± 63 | 224 ± 39 | 180 ± 29 | 182 ± 20 | 152 ± 12 |
| GRO/KC | 530 ± 139 | 422 ± 61 | 400 ± 49 | 369 ± 33 | 297 ± 22 | 321 ± 43* |
| MIP-1α | 137 ± 42 | 111 ± 34 | 87 ± 13 | 68 ± 8 | 71 ± 6 | 69 ± 6 |
| RANTES | 1264 ± 115 | 1162 ± 149 | 1176 ± 82 | 857 ± 59 | 783 ± 100 | 949 ± 74 |
| VEGF | 182 ± 47 | 187 ± 17 | 170 ± 21 | 134 ± 14* | 176 ± 26 | 130 ± 11* |
| MCP-1 | 2521 ± 535 | 2102 ± 319 | 1927 ± 122 | 1607 ± 103 | 1603 ± 225 | 1613 ± 92 |

Data are presented as mean ± standard error of the mean for 12 rats per group.

No significant differences were observed between the sham and vehicle groups for the cytokines shown.

**P* < 0.05, ***P* < 0.01 compared with vehicle group (Steel’s test).

i.v.: intravenous, p.o.: per os, IL: interleukin, GM-CSF: granulocyte-macrophage colony-stimulating factor, GRO/KC: growth-related oncogene/keratinocyte-derived chemokine, MIP: macrophage inflammatory protein, VEGF: vascular endothelial growth factor, MCP-1: monocyte chemoattractant protein-1
